# Prognostic Value of the Radiographic Assessment of Lung Edema Score in Mechanically Ventilated ICU Patients

**DOI:** 10.3390/jcm12041252

**Published:** 2023-02-04

**Authors:** Daan F. L. Filippini, Laura A. Hagens, Nanon F. L. Heijnen, Claudio Zimatore, Leila N. Atmowihardjo, Ronny M. Schnabel, Marcus J. Schultz, Dennis C. J. J. Bergmans, Lieuwe D. J. Bos, Marry R. Smit

**Affiliations:** 1 Department of Intensive Care, Amsterdam UMC, University of Amsterdam, 1105 AZ Amsterdam, The Netherlands; 2 Department of Intensive Care, Maastricht UMC+, Maastricht University, 6229 HX Maastricht, The Netherlands; 3 School of Nutrition and Translational Research in Metabolism (NUTRIM), Maastricht University, 6229 ER Maastricht, The Netherlands; 4 Department of Emergency and Organ Transplantation, University of Bari Aldo Moro, 70124 Bari, Italy; 5 Mahidol Oxford Tropical Medicine Research Unit (MORU), Mahidol University, Bangkok 10400, Thailand; 6 Nuffield Department of Medicine, University of Oxford, Oxford OX3 7BN, UK; 7 Department of Research and Development, Hamilton Medical AG, 7402 Bonaduz, Switzerland; 8 Department of Pulmonology, Amsterdam UMC, University of Amsterdam, 1105 AZ Amsterdam, The Netherlands; 9 Laboratory of Experimental Intensive Care and Anesthesiology (L.E.I.C.A.), University of Amsterdam, 1105 AZ Amsterdam, The Netherlands

**Keywords:** intensive care, critical care, mechanical ventilation, ARDS, prognostication, mortality, chest X-ray (CXR), pulmonary oedema, RALE score

## Abstract

Introduction: The Radiographic Assessment of Lung Edema (RALE) score provides a semi-quantitative measure of pulmonary edema. In patients with acute respiratory distress syndrome (ARDS), the RALE score is associated with mortality. In mechanically ventilated patients in the intensive care unit (ICU) with respiratory failure not due to ARDS, a variable degree of lung edema is observed as well. We aimed to evaluate the prognostic value of RALE in mechanically ventilated ICU patients. Methods: Secondary analysis of patients enrolled in the ‘Diagnosis of Acute Respiratory Distress Syndrome’ (DARTS) project with an available chest X-ray (CXR) at baseline. Where present, additional CXRs at day 1 were analysed. The primary endpoint was 30-day mortality. Outcomes were also stratified for ARDS subgroups (no ARDS, non-COVID-ARDS and COVID-ARDS). Results: 422 patients were included, of which 84 had an additional CXR the following day. Baseline RALE scores were not associated with 30-day mortality in the entire cohort (OR: 1.01, 95% CI: 0.98–1.03, *p* = 0.66), nor in subgroups of ARDS patients. Early changes in RALE score (baseline to day 1) were only associated with mortality in a subgroup of ARDS patients (OR: 1.21, 95% CI: 1.02–1.51, *p* = 0.04), after correcting for other known prognostic factors. Conclusions: The prognostic value of the RALE score cannot be extended to mechanically ventilated ICU patients in general. Only in ARDS patients, early changes in RALE score were associated with mortality.

## 1. Introduction

Acute respiratory distress syndrome (ARDS) is characterized by the acute onset of protein-rich alveolar edema resulting in hypoxemia [1,2]. However, a variable degree of pulmonary edema can be found in mechanically ventilated patients on the intensive care unit (ICU), with ARDS patients being on one extreme of the spectrum [3,4]. Recently, the Radiographic Assessment of Lung Edema (RALE) score was developed to semi-quantify lung edema by assessing the radiographic extent and density of alveolar opacities in each lung quadrant on a chest X-ray (CXR) [5]. The RALE score showed a correlation with gravimetric lung edema in explanted lungs [5] and was clinically mainly studied in patients with ARDS [6,7,8,9,10].

Pulmonary edema typically results in ventilation-perfusion mismatch, and subsequent hypoxemia may require ventilation with higher pressures due to decreased compliance of the lung [1]. At hospital presentation, baseline RALE scores demonstrated an association with ICU admission and mortality [11,12,13,14,15]. In the ICU, ARDS patients with higher baseline RALE scores, indicating more radiographic lung edema, were found to have a higher mortality in one study [5], although other studies found stronger associations between the change in RALE score and mortality [7,8,9]. In the ICU, the RALE score was primarily studied in patients with ARDS and remains understudied in less-selected ICU populations on mechanical ventilation. This is particularly relevant, as ARDS represents only a small subgroup of mechanically ventilated patients, and diagnosis is rather subjective and arbitrary [16,17].

In this study, we quantified the association between baseline levels of and early changes in RALE score and mortality in mechanically ventilated patients in the ICU. We hypothesized that the RALE score at baseline and early changes in RALE score over time are associated with mortality in mechanically ventilated ICU patients.

## 2. Methods

### 2.1. Study Design and Ethics

This is a secondary analysis performed within the ‘Diagnosis of Acute Respiratory Distress Syndrome’ (DARTS) project. The DARTS project is a prospective observational study that included mechanically ventilated patients in two university hospitals in The Netherlands (Amsterdam UMC, location AMC and Maastricht UMC+) between 03-2019 and 03-2021. The institutional review board of the Amsterdam UMC location AMC approved the study (W18_311 and 2018_287), and the complete study protocol was previously published [18]. Written deferred consent for use of data was obtained from patients and/or their legal representatives. For the present secondary analysis, CXR data was collected within 48 h after start of intubation (baseline) and at the day after inclusion (day 1).

### 2.2. Population

Patients were included if they were admitted to a participating ICU and expected to be mechanically ventilated for more than 24 h. Exclusion criteria were mechanical ventilation for more than 48 h in the 7 days prior to inclusion, a life expectancy shorter than 24 h at admission, tracheotomised patients and if breath sampling was deemed clinically inappropriate. For the present secondary analysis, only patients with a usable baseline CXR were eligible.

### 2.3. ARDS

The Berlin criteria were used to diagnose ARDS [19]. To minimize the inter-observer variability, an expert panel of three independent physicians scored the presence of ARDS based on clinical parameters, blood gas results and imaging (Appendix A) [20]. To allow for better comparisons with previous literature, patients with ARDS were further divided into ARDS distinctly caused by COVID-19 (COVID-ARDS) and ARDS not caused by COVID-19 (non-COVID-ARDS). Patients with cardiogenic pulmonary edema were scored as ‘no ARDS’.

### 2.4. RALE Scoring

The RALE score is composed of two aspects of pulmonary edema in four quadrants divided horizontally by a line through the first branch of the left main bronchus and vertically by the spinal column: consolidation extent, quantified as percentage of the CXR quadrant involved (0% = 0, <25% = 1, 25–50% = 2, 50–75% = 3, >75% = 4); and density (1 = hazy, 2 = moderate, 3 = dense). Consolidation extent and density are multiplied for each quadrant, and the total RALE score is obtained by addition of all quadrant scores, ranging from 0–48 [5]. First, one reviewer (D.F.L.F.) assessed the quality of all baseline and day 1 CXRs as ‘acceptable’, ‘borderline’ or ‘unusable’ based on general quality and possibly interfering comorbidities (e.g., pleural effusion, pneumothorax, subcutaneous emphysema). All CXRs with ‘acceptable’ quality were scored by one reviewer (D.F.L.F.) while validating the inter-observer agreement by using a 10% random sample scored by a second reviewer (L.N.A.). Images marked as ‘borderline’ or ‘unusable’ quality were assessed and scored by three reviewers (D.F.L.F., M.R.S. and L.N.A.) in a consensus meeting. All reviewers were trained by an expert (C.Z.) until an intraclass correlation coefficient (ICC) with the expert of >0.9 was reached. The reviewers are described in Appendix B and were blinded for all other patient data.

Early changes in RALE score (∆RALE) were calculated by subtracting the baseline RALE score from the day 1 RALE score, resulting in a negative ∆RALE when the RALE score decreased and a positive ∆RALE when the RALE score increased. Additionally, ∆RALE was dichotomized into greater than zero or not, indicating an increase in RALE score.

### 2.5. Outcomes

The primary endpoint was 30-day mortality, and the secondary endpoint was 90-day mortality. Endpoints were corrected for known prognostic variables in the general ICU population (age, gender, APACHE II score) and stratified for predefined subgroups (no ARDS, non-COVID-ARDS and COVID-ARDS) if the subgroups were sufficiently sized (*n* > 20). Additional outcomes were the difference in baseline RALE scores between predefined subgroups and the association between baseline RALE score and ARDS severity.

### 2.6. Statistical Analysis

Continuous data were expressed as mean with the standard deviation or median with the interquartile range according to statistical distribution (assessed using histograms, Q-Q plots and the Shapiro–Wilk test). Depending on distribution, differences between continuous variables were analysed using a *t*-test, a Mann–Whitney-U or Kruskal–Wallis test. Categorical data were expressed as numbers and percentages, and differences were tested using the Chi-square test or Fisher exact test. The primary and secondary endpoints were analysed using logistic regression with and without other known prognostic variables (age, gender, APACHE II score) [5,21]. The relationship between RALE score and mortality was visualised using locally estimated scatterplot smoothing (LOESS) regression in order to see if categorisation using arbitrary cut-off values (e.g., quartiles) was feasible. The correlation between continuous variables was plotted using scatterplots and assessed using Pearson or Spearman correlation coefficients. The RALE score reproducibility was assessed by calculating inter-observer agreement using the intraclass correlation coefficient (ICC). Tests were two-sided, with a type I error set at 5%. Statistical analyses were performed using RStudio, version 4.0.3 (R Foundation for Statistical Computing, Vienna, Austria).

## 3. Results

### 3.1. Study Population

**Figure 1:**
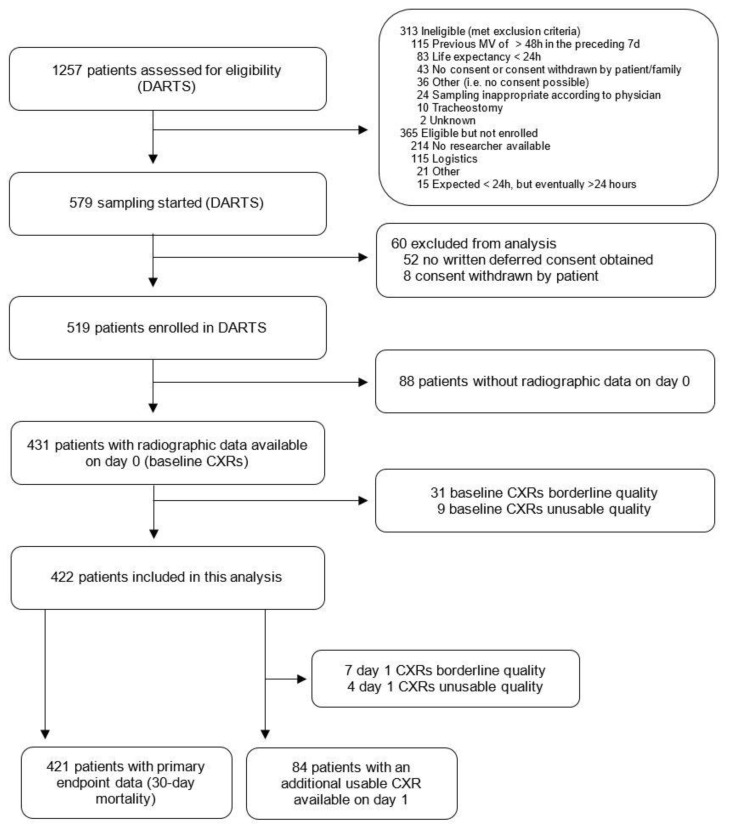
Flowchart of the inclusion process. DARTS = ‘Diagnosis of Acute Respiratory Distress Syndrome’ project [18]; MV = Mechanical Ventilation; CXR = Chest X-ray.

A total of 422 (81%) of the 519 patients included in the DARTS project had a usable baseline CXR available and were included in the present study (Figure 1, Table 1 and Table S1). ARDS was present in 157 (37%) of the included patients, with COVID-19 accounting for 50 (32%) of the cases. Out of 422 patients, 153 (36%) died by day 30. Patients who did not survive were older, were more frequently admitted for emergency surgery, had higher APACHE II and SOFA scores and a lower PaO_2_/FiO_2_ (Table 1). COVID-ARDS and non-COVID-ARDS were combined into one ARDS subgroup when looking at ∆RALE because additional CXRs were available for 5 COVID-ARDS patients only.

### 3.2. RALE Scoring

**Figure 2:**
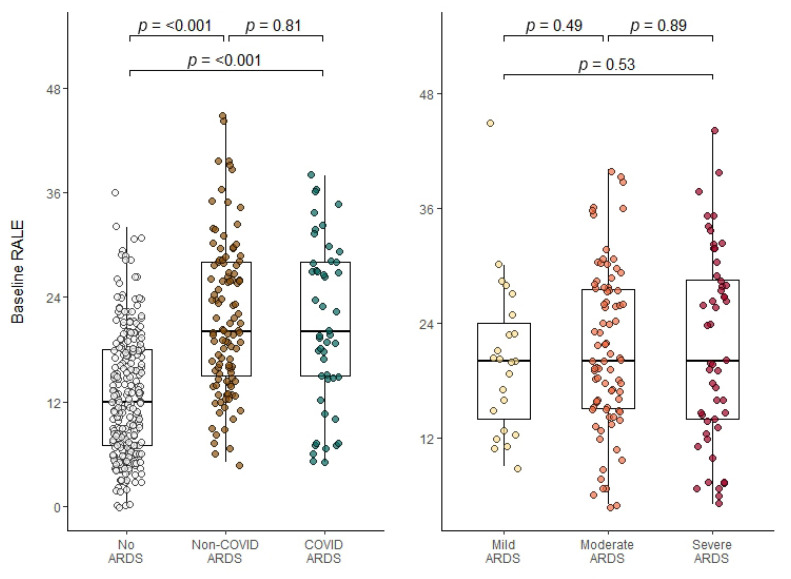
Distribution of baseline RALE scores. Data are stratified and coloured by predefined ARDS categories (**left**) and ARDS severity (**right**). Individual data points are displayed as dots. Distributions of the different groups are compared by means of a Mann–Whitney U test, which is displayed above the boxplots. RALE = Radiographic Assessment of Lung Edema; ARDS = Acute Respiratory Distress Syndrome; COVID = Coronavirus disease 2019.

There was good inter-observer agreement between the reviewers (ICC = 0.782, 95% CI: 0.65–0.87). Of the 519 evaluated baseline and day 1 CXRs, 51 (9.8%) with doubtful quality were discussed in a consensus meeting. Of these, 38 (75%) could be scored and 13 (26%) were deemed ‘unusable’ and could not be scored. The median baseline RALE score was 15 (IQR 8–21) (Table S2). In patients without ARDS, the median baseline RALE score of 12 (IQR 7–18) was lower than in COVID-ARDS (20, [IQR 15–28], *p* < 0.001, Figure 2, Table S2) and non-COVID-ARDS (20, (IQR 15–28), *p* < 0.001, Figure 2). There was no difference in baseline RALE scores between COVID-ARDS and non-COVID-ARDS (*p* = 0.81, Figure 2). Baseline RALE score correlated with PaO_2_/FiO_2_ (r = −0.44, *p* < 0.001, Figure S1) in the total population, but not when only patients with ARDS were considered (r = −0.03, *p* = 0.68, Figure 2 and Figure S1).

### 3.3. Association between Baseline RALE and Mortality

**Figure 3:**
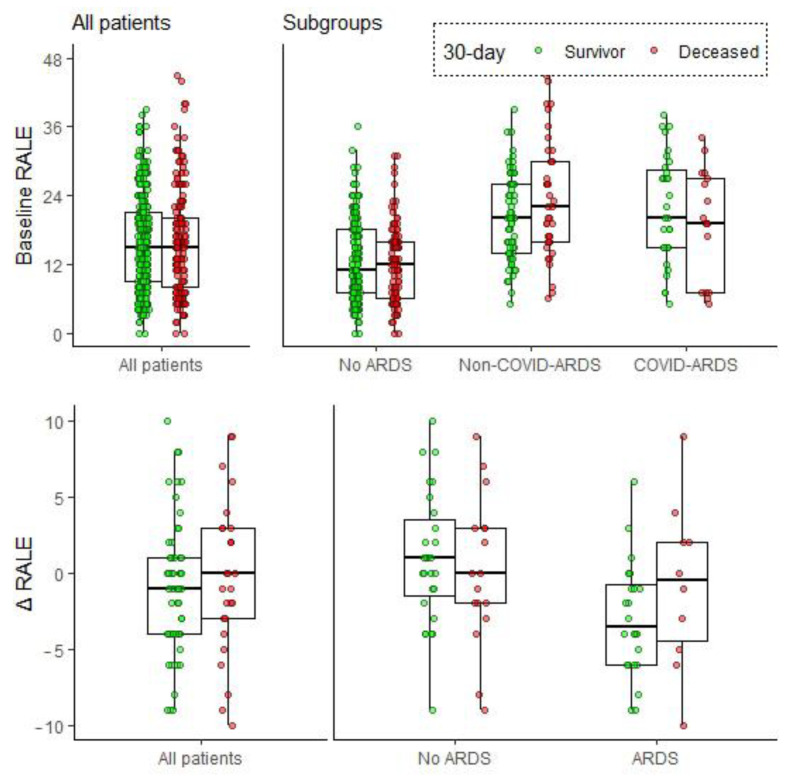
Baseline RALE scores and ∆RALE. Data are stratified and coloured by 30-day mortality in the entire study cohort and in predefined ARDS subgroups (no ARDS, non-COVID-ARDS, COVID-ARDS). As there were only 5 COVID-ARDS patients with an additional day 1 CXR available, COVID-ARDS and non-COVID-ARDS were combined into ARDS when looking at ∆RALE. Individual patient data points are plotted as dots. RALE = Radiographic Assessment of Lung Edema; ARDS = Acute Respiratory Distress Syndrome; ∆RALE = Early change in RALE score (RALE day 1—RALE baseline); COVID = Coronavirus disease 2019.

Baseline RALE was not significantly associated with 30-day mortality in the entire study cohort or in predefined subgroups, in univariate analysis or after correcting for known prognostic factors (Table 2, Figure 3). In terms of 90-day mortality, a positive association was found only in non-COVID-ARDS patients (1.06 OR increase per increment baseline RALE, 95% CI: 1.01–1.11, *p* = 0.015, Figure S2, Table S3). Adjusting for known prognostic factors (age, gender, APACHE II) did not alter these results. A visual assessment of the relation between RALE and mortality did not result in any cut-off that would increase the prognostic value of the RALE score; therefore, we refrained from using arbitrary cut-offs to dichotomize this variable in additional analyses (Figure S3).

### 3.4. Early Changes in RALE Score and Survival

∆RALE was not associated with 30-day mortality in the entire study cohort. In ARDS patients, however, ∆RALE was associated with 30-day mortality after correcting for other prognostic factors (1.21 OR increase per ∆RALE increment, 95% CI 1.02–1.51, *p* = 0.046, Table 3). A ∆RALE above zero (indicating an increase in RALE score between baseline and day 1) was also associated with an increased 30-day mortality in ARDS patients (OR 17.38, 95% CI 1.72–422.39, *p* = 0.034, Table 3).

∆RALE had no association with 90-day mortality in the entire study cohort and predefined ARDS subgroups, and adjusting for confounders did not change the results (Figure S2, Table S4). A ∆RALE above zero was also not associated with 90-day mortality (Table S4).

## 4. Discussion

In this study, we assessed the prognostic capacity of the RALE score in mechanically ventilated ICU patients. The main finding was that baseline RALE score was not associated with 30-day mortality in our cohort of mechanically ventilated ICU patients or in a subgroup of ARDS patients. ∆RALE scores (changes from baseline to day 1) were associated with mortality in ARDS patients, although not in mechanically ventilated ICU patients in general.

We found no associations between the baseline RALE score and mortality in mechanically ventilated ICU patients. In subgroups of ARDS patients, we did not find associations between ARDS subgroups and our primary outcome, 30-day mortality, but did find an association between the baseline RALE score and 90-day mortality in non-COVID-ARDS patients only. These conflicting results are in line with previous literature, as most studies found no associations [8,9,10,11], whereas the original [5] and another study [7] did find an association between baseline RALE scores and mortality in ARDS patients. These inconsistent findings in ARDS studies might be caused by the heterogeneous ARDS population, the different inclusion and exclusion criteria or the timing of the CXRs [5,7,8,9,10,11]. For example, the original RALE study [5] specifically excluded patients with comorbidities which might lead to impaired assessment of pulmonary edema. Taken together, there is conflicting evidence for associations between baseline RALE score and mortality in ARDS patients, and our results imply that extension to mechanically ventilated ICU patients in general is not possible.

The results of the present study confirmed the universally reported prognostic value of ∆RALE score in ARDS patients, despite our short one-day follow-up period for the second CXR. The consistency of the association between ∆RALE score and mortality in ARDS patients increases the validity of the score and is remarkable, as studies have methodological differences with different time intervals, and the ARDS population is heterogeneous as previously mentioned [7,8,9]. This heterogeneity may also be the cause for the absence of an association ∆RALE and 90-day mortality in our study. Our findings do suggest that the prognostic capacity of the ∆RALE score is limited to patients with pulmonary edema and severe respiratory failure and cannot be extended to all mechanically ventilated ICU patients.

The RALE score was found to be associated with different indicators of lung damage such as biomarkers SP-D, RAGE, IL-6 and sTNFR1; lung injury scores; and compliance [7,8,11,23]. However, previous studies showed disagreement on the association between RALE scores and PaO_2_/FiO_2_ [7,8,9,11]. In our study cohort of mechanically ventilated ICU patients, we found an association between baseline RALE and PaO_2_/FiO_2_, but this could not be replicated in a subgroup of ARDS patients only. Based on these findings, we speculate that even though the RALE score and PaO_2_/FiO_2_ are both abnormal in ARDS when compared to no ARDS, they are not sufficiently associated within ARDS patients, as in these patients, other processes contribute to deterioration of gas-exchange besides the quantity of radiographic pulmonary edema.

The inter-observer agreement of the RALE score in the present study was high, although slightly lower than in previous ARDS studies [5,6,7,8]. This difference might be caused by the large group of non-ARDS patients in our cohort, which have varying causes, types and levels of pulmonary edema. Nonetheless, the agreement was still good, and our results in subgroups of ARDS patients were in line with previous studies. These findings further add to the reproducibility of the RALE score, and this, together with the simplicity and routine bedside availability, suggests a role for the RALE score in radiographic lung edema quantification [24,25]. An interesting alternative could be lung ultrasound, which is increasingly used and can also semi-quantify lung edema at bedside [26,27,28]. A CT scan might be a less attractive alternative, as it is less available, more costly, has a higher radiation dose and requires transport outside of the ICU [29,30].

Limitations of the present study include the possibility of selection bias regarding the ∆RALE, as only patients selected for a second CXR had a ∆RALE score available. Information about the practice methods when selecting patients for a second CXR was unavailable. Other limitations concerning the ∆RALE were the short time span of only one day between CXRs and the small subgroup sizes. Regardless, we were able to find a significant association with mortality in patients with ARDS. Finally, because of the observational study design, CXR acquisition and PEEP strategies were not strictly protocolized, which might have resulted in slight time variability and impaired assessment of lung edema [31].

The present study has several strengths, with the most prominent being the large multicentre study cohort of consecutive ICU patients. As the DARTS project had few limiting exclusion criteria, the study cohort consisted of a broad range of mechanically ventilated patients, including ARDS patients with different aetiologies. The availability of baseline CXRs was high, and the loss to follow-up was minimal, limiting the possibility of selection bias. This is the first study to suggest that the RALE score has no prognostic value in mechanically ventilated ICU patients in general. Because of the small sample size in our ∆RALE non-ARDS subgroup and the limited published data on RALE in this patient group, further research is warranted to confirm this absence of prognostic value. In ARDS patients, our findings together with the existing body of literature on the ∆RALE score suggest that sequential CXR scoring can inform clinicians of prognosis. Future studies could focus on standardizing the time interval and quantification method of the ∆RALE score in ARDS patients and may subsequently investigate the potential of the RALE score to select subgroups of ARDS patients that show similar response to treatment strategies.

In conclusion, prognostic associations with RALE scores were not found in a general population of mechanically ventilated ICU patients. Only in ARDS patients, there was an evident association between ∆RALE and mortality.

## Figures and Tables

**Table 1:** Baseline characteristics.

	All *n* = 422	Survived at 30 Days *n* = 268	Deceased at 30 Days *n* = 153	*p*-Value
**Demographics**				
Age (years (SD))	62 (14)	60 (14)	66 (12)	<0.001
Male (%)	290 (68.7)	189 (70.5)	100 (65.4)	0.323
BMI (kg m^−2^)	26.1 (23.4, 29.9)	26.1 (23.6, 29.4)	26.3 (22.9, 30.2)	0.811
**Admission characteristics**				
ICU stay inclusion (days)	1 (0, 1.75)	1 (0, 2)	1 (0, 1)	0.298
Admission type (%)				0.007
*- Medical*	307 (72.7)	195 (72.8)	2.5)	
*- Emergency surgical*	60 (14.2)	30 (11.2)	30 (19.6)	
*- Planned surgical*	55 (13.0)	43 (16.0)	12 (7.8)	
COVID-19 (%)	50 (11.8)	33 (12.3)	17 (11.1)	0.834
**Respiratory**				
Maximum airway pressure (cmH_2_O)	21 (16, 26)	21 (16, 26)	21 (17, 27)	0.655
Driving Pressure (cmH_2_O)	14 (9, 18)	14 (9, 18)	14 (10, 18)	0.651
PEEP (cmH_2_O)	8 (5, 10)	8 (5, 10)	8 (5, 10)	0.625
**Severity**				
ARDS category (%)				0.997
*- No ARDS*	265 (62.8)	169 (63.1)	96 (62.7)	
*- Mild ARDS*	23 (5.5)	15 (5.6)	8 (5.2)	
*- Moderate ARDS*	83 (19.7)	52 (19.4)	30 (19.6)	
*- Severe ARDS*	51 (12.1)	32 (11.9)	19 (12.4)	
APACHE II score	20 (15, 25)	20 (15, 24)	23 (18, 26)	<0.001
SOFA score	9 (7, 11)	9 (7, 11)	10 (7, 11)	0.043
PaO_2_/FiO_2_ (mmHg)	179 (113, 270)	188 (119, 287)	154 (109, 244)	0.040
Lactate (mmol/L)	1.6 (1.2, 2.5)	1.60 (1.1, 2.2)	1.90 (1.3, 3.3)	<0.001
**Outcomes**				
ICU length of stay (days)	7 (3, 13)	7 (3, 13)	6 (3, 12.25)	0.237
ICU mortality (%)	132 (32.5)	5 (1.9)	127 (84.7)	<0.001

Data are stratified by 30-day mortality. SD = Standard Deviation; BMI = Body Mass Index; ICU = Intensive Care Unit; PEEP = Positive End-Expiratory Pressure; ARDS = Acute Respiratory Distress Syndrome; APACHE II = Acute Physiology and Chronic Health Evaluation II [21]; SOFA = Sequential Organ Failure Assessment [22].

**Table 2:** Baseline RALE scores and 30-day mortality.

	All Patients *n* = 421		No ARDS *n* = 265		Non-COVID-ARDS *n* = 108		COVID-ARDS *n* = 48	
	OR (CI)	*p*-Value	OR (CI)	*p*-Value	OR (CI)	*p*-Value	OR (CI)	*p*-Value
**Univariable analyses**								
Baseline RALE	1.01 (0.98–1.03)	0.66	0.99 (0.96–1.03)	0.77	1.04 (1–1.1)	0.07	0.97 (0.91–1.03)	0.37
**Multivariable analyses (adjusted for confounders: age, gender APACHE II)**								
Baseline RALE	1 (0.98–1.02)	0.92	0.99 (0.95–1.02)	0.47	1.05 (1–1.1)	0.07	0.99 (0.91–1.07)	0.80

Data are displayed for all patients and predefined subgroups (no ARDS, non-COVID-ARDS, COVID-ARDS). Values are derived by logistic regression and are displayed as OR (95% CI) increase per 1 point increment of predictor variable. RALE = Radiographic Assessment of Lung Edema; OR = Odds Ratio; CI = Confidence Interval; ARDS = Acute Respiratory Distress Syndrome; COVID = Coronavirus disease 2019.

**Table 3:** ∆RALE and 30-day mortality.

	All Patients *n* = 84		No ARDS *n* = 48		ARDS *n* = 36	
	OR (CI)	*p*-Value	OR (CI)	*p*-Value	OR (CI)	*p*-Value
**Univariable analyses**						
∆RALE *	1 (0.93–1.07)	0.98	0.97 (0.88–1.07)	0.55	1.02 (0.92–1.14)	0.64
∆RALE > 0	1.26 (0.44–3.54)	0.64	0.64 (0.16–2.42)	0.56	3.51 (0.5–25.74)	0.18
**Multivariable analyses (corrected for confounders)**						
∆RALE *	1.02 (0.94–1.1)	0.64	0.97 (0.87–1.08)	0.60	1.21 (1.02–1.51)	0.04
∆RALE > 0	1.32 (0.49–3.56)	0.58	0.61 (0.17–2.09)	0.44	17.38 (1.72–42.39)	0.03

Data are displayed for all patients and predefined subgroups. As the number of COVID patients with day 1 data available was low (n = 5), COVID-ARDS and non-COVID-ARDS were combined into ARDS. Values are displayed as OR (95% CI) and are derived by means of Fisher exact test (categorical predictor) or logistic regression (continuous/mixed predictors). * = OR represents increase per 1- point increment of predictor. RALE = Radiographic Assessment of Lung Edema; ∆RALE = Early changes in RALE score (RALE day 1-RALE baseline); OR = Odds Ratio; CI = Confidence Interval; ARDS = Acute Respiratory Distress Syndrome; COVID = Coronavirus disease 2019.

## Data Availability

The datasets used and/or analysed during the current study are available from the corresponding author on reasonable request.

## Supplementary Materials

The following supporting information can be downloaded at https://www.mdpi.com/article/10.3390/jcm12041252/s1. Table S1: Admission characteristics stratified by ARDS subgroup. Table S2: Distribution of baseline RALE scores. Figure S1: Correlation plots of baseline RALE and PaO_2_/FiO_2_. Figure S2: Baseline RALE scores and ∆RALE (90-day mortality). Table S3: Baseline RALE (90-day mortality). Figure S3a: Baseline RALE score and 30-day mortality. Figure S3b: Baseline RALE score and 90-day mortality. Table S4: ∆RALE and 90d mortality.

## Abbreviations

∆RALE = Early changes in RALE score; APACHE II = Acute Physiology and Chronic Health Evaluation II [21]; ARDS = Acute Respiratory Distress Syndrome; BMI = Body Mass Index; CI = Confidence Interval; COVID = Coronavirus disease 2019; COVID-ARDS = ARDS caused by COVID; CXR = Chest X-ray; DARTS = Diagnosis of Acute Respiratory Distress syndrome (project); ICC = Intraclass Correlation Coefficient; ICU = Intensive Care Unit; IQR = Interquartile Range; LOESS = Locally Estimated Scatterplot Smoothing; Non-COVID-ARDS = ARDS not caused by COVID; OR = Odds Ratio; PEEP = Positive End-Expiratory Pressure; RALE = Radiographic Extent of Lung Edema; SD = Standard Deviation; SOFA = Sequential Organ Failure Assessment [22].

## Appendix A. ARDS Scoring Method

Diagnosis of ARDS is known to be challenging, mainly due to the high inter-observer variability in interpretation of chest imaging [16,32]. To improve agreement for the presence of bilateral opacities, an expert panel (three independent physicians) scored each chest X-ray (CXR) and computed tomography (CT) scan on an 8-point scale for confidence of ARDS. These scores were subsequently transformed into a decision matrix with three options: (1) ‘No ARDS’ if sufficient certainty was deemed to exclude ARDS, (2) ‘ARDS’ if sufficient certainty was deemed to diagnose ARDS and (3) ‘uncertain diagnosis’ in case of general uncertainty or conflicting results.

The decision matrix from the CT-scan was used if this resulted in a unanimous decision for or against ARDS. If the CT-scan’s decision matrix was inconclusive (option 3), the decision matrix for the CXR was used. If uncertainty remained after using both CT-scan and CXR decision matrices, the patient was classified as uncertain. Uncertain patients were discussed in a consensus meeting, where they were classified as ‘likely ARDS’ or ‘likely no ARDS’.

Detailed explanation of the scoring process, classification algorithm and figures are presented elsewhere [18].

## Appendix B. Characteristics of CXR Scorers

**Name**	**Title**	**Current Role**
D.F.L.F.	Medical Doctor	PhD-student
L.N.A.	Medical Doctor	PhD-student
M.R.S.	PhD, Technical Physician	Postdoctoral researcher
C.Z.	Anaesthesiologist, Intensivist	Intensivist, PhD-student
