# Supplementary material for: Prognostic Value of the Radiographic Assessment of Lung Edema Score in Mechanically Ventilated ICU Patients
Source: J Clin Med. 2023 Feb 4;12(4):1252. doi: 10.3390/jcm12041252 (PMC9960783; doi:10.3390/jcm12041252)
Supplement: Supplementary file 1 [file jcm-12-01252-s001.zip › jcm-2149384-supplementary.pdf]

Prognostic Value of the Radiographic Assessment of Lung Edema Score in Mechanically Ventilated ICU Patients

Daan F.L. Filippini, Laura A. Hagens, Nanon F.L. Heijnen, Claudio Zimatore, Leila N. Atmowihardjo, Ronny M. Schnabel, Marcus J. Schultz, Dennis C.J.J. Bergmans, Lieuwe D.J. Bos, Marry R. Smit

Supplementary Materials

Table of contents

Table S1. Admission characteristics stratified by ARDS 1

Table S2. Distribution of baseline RALE scores 2

Figure S1. Correlation plots of baseline RALE and PaO_2 / FiO_2 3

Figure S2. Baseline RALE scores and ∆RALE (90-day mortality). 4

Table S3. Baseline RALE (90-day mortality) 5

Figure S3a. Baseline RALE score and 30-day mortality. 6

Figure S3b. Baseline RALE score and 90-day mortality 6

Table S4. ∆RALE and 90d mortality 7

Table S1. Admission characteristics stratified by ARDS subgroup.

	**No ARDS**	**Non-COVID-ARDS**	**COVID-ARDS**	p
n	265	109	48	
**Demographics**				
Age (years, mean (SD))	61.66 (14.86)	63.03 (12.95)	63.54 (11.20)	0.546
Male (%)	179 (67.5)	78 (71.6)	33 (68.8)	0.749
BMI (kg m^{-2})	25.99 [22.87, 29.39]	26.15 [23.57, 29.47]	27.92 [24.90, 33.06]	0.014
**History**				
COPD (%)	28 (10.6)	8 (7.3)	2 (4.2)	0.282
Chronic respiratory failure (%)	2 (0.8)	3 (2.8)	0 (0.0)	0.194
Renal failure (%)	22 (8.3)	6 (5.5)	3 (6.2)	0.612
Cardiac failure (%)	45 (17.0)	4 (3.7)	4 (8.3)	0.001
**Admission type**				
Admission type (%)				<0.001
Emergency surgical	46 (17.4)	13 (11.9)	1 (2.1)	
Medical	176 (66.4)	84 (77.1)	47 (97.9)	
Planned surgical	43 (16.2)	12 (11.0)	0 (0.0)	
Neurosurgery (%)	45 (17.0)	6 (5.5)	0 (0.0)	<0.001
High risk surgery (%)				<0.001
Acute abdomen	8 (8.9)	7 (26.9)	0 (0.0)	
Aortic vascular	24 (26.7)	5 (19.2)	0 (0.0)	
Cardiac	22 (24.4)	3 (11.5)	0 (0.0)	
Other	34 (37.8)	10 (38.5)	0 (0.0)	
**Admission characteristics**				
Active malignancy (%)	24 (9.1)	21 (19.3)	4 (8.3)	0.015
Immunoincompetence (%)	13 (4.9)	8 (7.3)	3 (6.2)	0.642
Shock (%)	27 (10.2)	11 (10.1)	1 (2.1)	0.191
Extrapulmonary sepsis (%)	29 (10.9)	19 (17.4)	1 (2.1)	0.019
Pancreatitis (%)	3 (1.1)	4 (3.7)	0 (0.0)	0.138
Chemotherapy (%)	8 (3.0)	6 (5.5)	2 (4.2)	0.514
Aspiration (%)	22 (8.3)	16 (14.7)	1 (2.1)	0.029
**Respiration at inclusion**				
FiO_2 (%, median [IQR])	30 [25, 40]	40 [31, 55]	51 [44, 69]	<0.001
PEEP (cmH_2O)	7 [5, 8]	10 [8, 12]	10 [10, 12]	<0.001
TV per IBW (mL kg^{-1})	7.05 [6.15, 8.53]	7.24 [6.10, 8.63]	7.21 [6.03, 8.56]	0.837
Respiratory Rate (min^{-1})	18 [15, 22]	20 [16, 26]	21 [16, 26]	<0.001
Driving Pressure (cmH_2O)	13 [9, 17]	16 [10, 19]	15 [9, 18]	0.004
**Severity**				
PaO_2 / FiO_2 (mmHg)	233 [154, 325]	126 [97, 180]	105 [80, 137]	<0.001
APACHE II score	20 [15, 26]	20 [15, 25]	18.50 [13, 22]	0.040
ECMO (%)	18 (6.8)	3 (2.8)	0 (0.0)	0.064
SOFA score	9 [7, 11]	9 [7, 12]	7 [6, 9]	<0.001

Data is expressed as median [IQR], except for categorical variables (mean (%)) and age (mean (SD)). BMI = Body Mass Index, COPD = Chronic Obstructive Pulmonary Disease, PEEP = Positive End-Expiratory Pressure, TV = Tidal Volume, IBW = Ideal Body Weight, ECMO = Extracorporeal Membrane Oxygenation

Table S2. Distribution of baseline RALE scores

	n	Baseline RALE
Entire study cohort	422	15 [8, 21]
No ARDS	265	12 [7, 18]
Non-COVID-ARDS	127	20 [15, 28]
COVID-ARDS	63	20 [15, 28]
Mild ARDS	23	20 [14, 24]
Moderate ARDS	83	20 [15, 27.5]
Severe ARDS	51	20 [14, 28.5]

Data are displayed as median [IQR]. Data are displayed for the entire study cohort and for different patient subgroups.

Figure S1. Correlation plots of baseline RALE and PaO_2 / FiO_2. In all patients (left) and ARDS patients only (right).

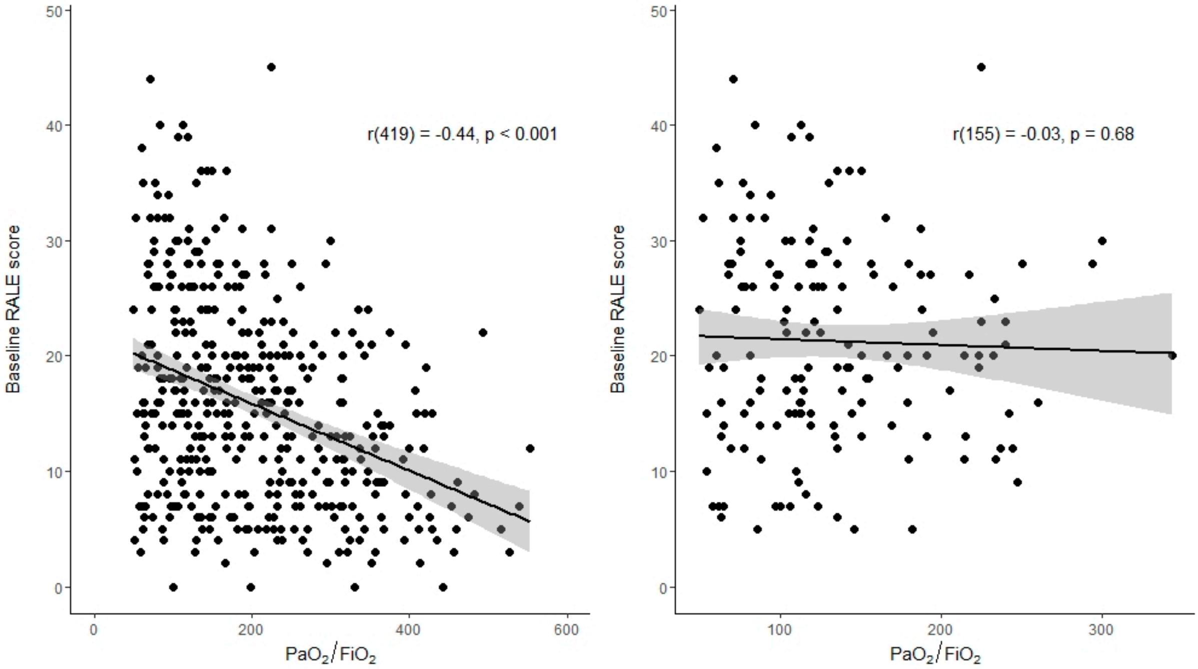

Individual patient data points are displayed as dots. On the x-axis, PaO_2 / FiO_2 is displayed in mmHg and on the y-axis the corresponding baseline RALE scores. The annotated text represents Pearson correlation coefficients and are displayed as “r(n) = correlation coefficient, p-value”

Figure S2. Baseline RALE scores and ∆RALE (90-day mortality).

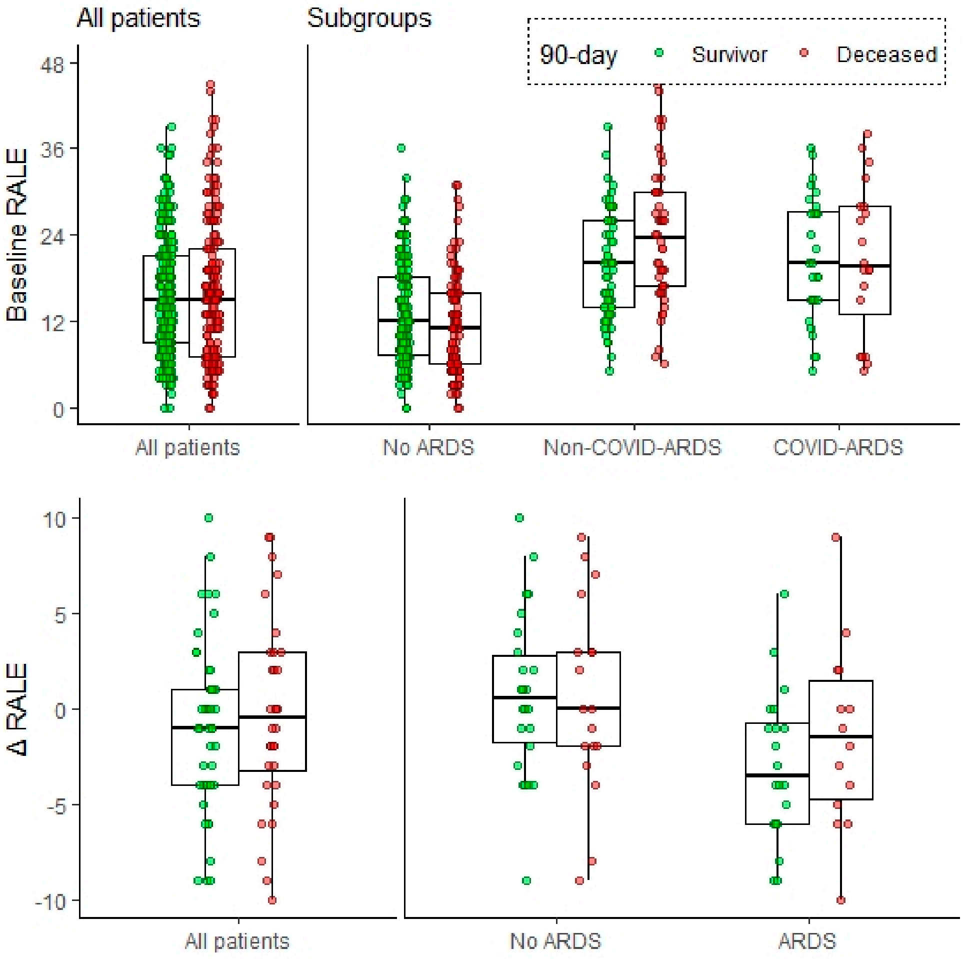

Data are stratified and colored by 90-day mortality in the entire study cohort and in predefined ARDS subgroups (ARDS COVID, ARDS non-COVID, no ARDS). As there were only 5 COVID-ARDS patients with an additional day 1 CXR available, COVID-ARDS and non-COVID-ARDS were combined into ARDS when looking at ∆RALE. Individual patient data points are plotted as dots. RALE = radiographic assessment of lung edema, ARDS = acute respiratory distress syndrome, ∆RALE = early change in RALE score (RALE day 1 – RALE baseline).

Table S3. Baseline RALE (90-day mortality)

	All patients n = 422		No ARDS n = 265		non-COVID ARDS n = 109		COVID ARDS n = 48	
	HR (CI)	p	HR (CI)	p	HR (CI)	p	HR (CI)	p
**Univariable analsyses**								
Baseline RALE*	1.01 (0.98 - 1.03)	0.542	0.97 (0.94 - 1.01)	0.139	1.06 (1.01 - 1.11)	0.015	1 (0.94 - 1.06)	0.971
**Multivariable analyses (adjusted for confounders: age, gender Apache II)**								
Baseline RALE*	1 (0.98 - 1.03)	0.713	0.96 (0.93 - 1)	0.061	1.06 (1.01 - 1.12)	0.014	1.02 (0.94 - 1.1)	0.64

Data are displayed for all patients and predefined subgroups (No ARDS, non-COVID-ARDS, COVID-ARDS). Values are derived by logistic regression and are displayed as OR (95% CI) increase per 1 point increment of predictor variable. RALE = Radiographic assessment of lung edema, OR = Odds ratio, CI = Confidence interval, ARDS = Acute respiratory distress syndrome, COVID = Coronavirus disease 2019.

Figure S3a. Baseline RALE score and 30-day mortality.

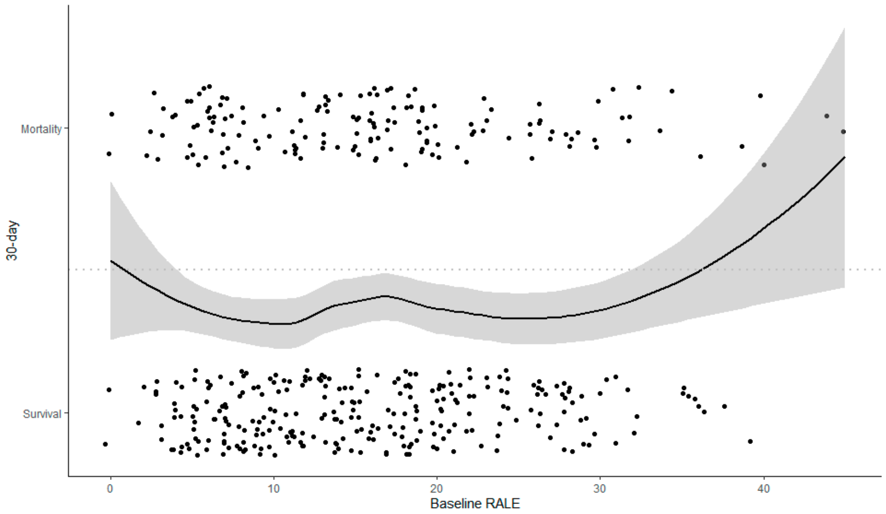

Individual patient data points are displayed as dots. On the x-axis, baseline RALE scores are displayed and on the y-axis 30-day mortality is jittered. The black trend line is derived by means of LOESS regression, with the 95% CI in gray.

Figure S3b. Baseline RALE score and 90-day mortality

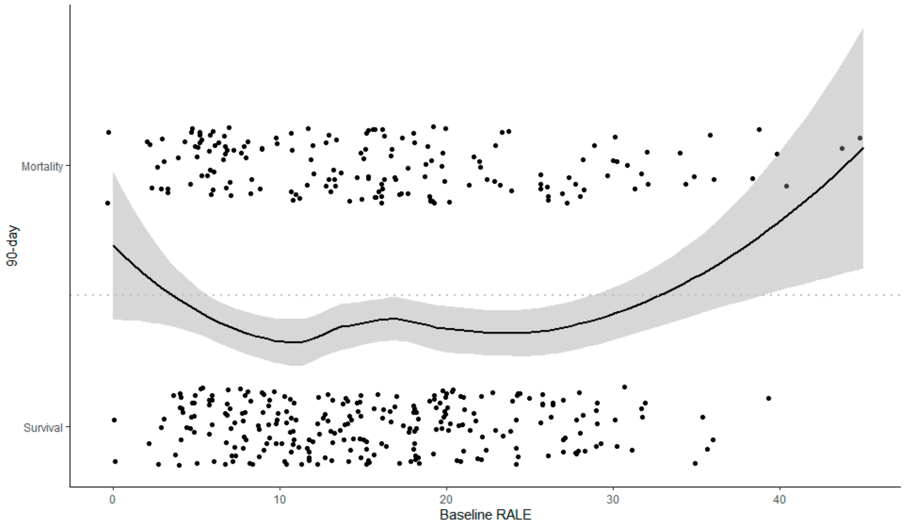

Individual patient data points are displayed as dots. On the x-axis, baseline RALE scores are displayed and on the y-axis 30-day mortality is jittered. The black trend line is derived by means of LOESS regression, with the 95% CI in gray.

Table S4. ∆RALE and 90d mortality

	**All patients** n = 84		**No ARDS** n = 48		**ARDS** n = 36	
	HR (CI)	p	HR (CI)	p	HR (CI)	p
**Univariable analyses**						
∆RALE*	1 (0.93 - 1.07)	0.995	0.99 (0.9 - 1.09)	0.837	1.01 (0.91 - 1.12)	0.845
∆RALE > 0	1.05 (0.38 – 2.86)	1	0.78 (0.21 – 2.9)	0.771	1.77 (0.27 – 11.85)	0.683
**Multivariable analyses (corrected for confounders)**						
∆RALE *	1.02 (0.94 - 1.09)	0.677	0.99 (0.89 - 1.1)	0.882	1.14 (0.99 - 1.34)	0.079
∆RALE > 0	1.07 (0.41 - 2.78)	0.895	0.76 (0.21 - 2.62)	0.661	4.26 (0.62 - 39.01)	0.158

Data are displayed for all patients and predefined subgroups. As the number of COVID patients with day 1 data available was low (n=5), COVID-ARDS and Non-COVID-ARDS were combined into ARDS. Values are displayed as OR (95% CI), and are derived by means of Fisher exact test (categorical predictor) or logistic regression (continuous/mixed predictors). * = OR represents increase per 1 point increment of predictor. RALE = Radiographic assessment of lung edema, ∆RALE = Early changes in RALE score, OR = Odds ratio, CI = Confidence interval, ARDS = Acute respiratory distress syndrome, COVID = Coronavirus disease 2019.
